# PtNi Alloy Cocatalyst Modification of Eosin Y-Sensitized g-C_3_N_4_/GO Hybrid for Efficient Visible-Light Photocatalytic Hydrogen Evolution

**DOI:** 10.1186/s11671-018-2448-y

**Published:** 2018-02-02

**Authors:** Peng Wang, Lanlan Zong, Zhongjie Guan, Qiuye Li, Jianjun Yang

**Affiliations:** National & Local Joint Engineering Research Center for Applied Technology of Hybrid Nanomaterials, Henan University, Kaifeng, 475004 China

**Keywords:** PtNi alloy cocatalyst, g-C_3_N_4_/GO, Eosin Y-sensitization, Hydrogen evolution

## Abstract

An economic and effective Pt-based alloy cocatalyst has attracted considerable attention due to their excellent catalytic activity and reducing Pt usage. In this study, PtNi alloy cocatalyst was successfully decorated on the g-C_3_N_4_/GO hybrid photocatalyst via a facile chemical reduction method. The Eosin Y-sensitized g-C_3_N_4_/PtNi/GO-0.5% composite photocatalyst yields about 1.54 and 1178 times higher hydrogen evolution rate than the Eosin Y-sensitized g-C_3_N_4_/Pt/GO-0.5% and g-C_3_N_4_/Ni/GO-0.5% samples, respectively. Mechanism of enhanced performance for the g-C_3_N_4_/PtNi/GO composite was also investigated by different characterization, such as photoluminescence, transient photocurrent response, and TEM. These results indicated that enhanced charge separation efficiency and more reactive sites are responsible for the improved hydrogen evolution performance due to the positive synergetic effect between Pt and Ni. This study suggests that PtNi alloy can be used as an economic and effective cocatalyst for hydrogen evolution reaction.

**Graphical abstract:**
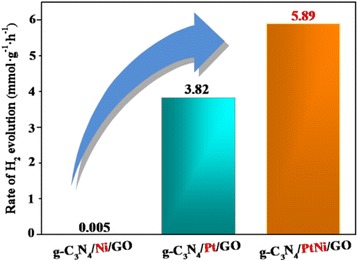
A significant enhancement of photocatalytic H_2_ evolution is realized over the Eosin Y-sensitized g-C_3_N_4_/PtNi/GO composite with PtNi alloy as an efficient cocatalyst.

A significant enhancement of photocatalytic H_2_ evolution is realized over the Eosin Y-sensitized g-C_3_N_4_/PtNi/GO composite with PtNi alloy as an efficient cocatalyst.

## Background

Sustainable and large-scale hydrogen evolution from water using solar energy is considered to be one of the promising method toward solving the energy crisis and environmental pollution [1, 2]. To achieve this goal, a visible-light response photocatalyst and an efficient cocatalyst are required [3–5]. Usually, loading noble metal Pt as an efficient cocatalyst is highly necessary for achieving a high hydrogen evolution rate [6–8]. However, Pt is rare and expensive, which hinders its practical application. Reducing the amount of Pt usage while simultaneously maintaining its excellent catalytic activity for hydrogen evolution is desired. Replacing part of Pt with transition metal (Ni, Co, Cu, Fe, etc.) to form a bimetallic alloy cocatalyst is a promising potential way for achieving excellent catalytic activity and reducing the use of Pt [9–11]. In some case, the catalytic performance of Pt-based bimetallic alloy cocatalyst is comparable with pure Pt due to the positive synergetic effect between the two metals. Therefore, a visible-light response photocatalyst loaded with a bimetallic alloy cocatalyst has been paid more attention in recently.

Yu et al. reported that PtCo or PtNi alloy cocatalyst-modified Cu_2_ZnSnS_4_ showed higher H_2_ production efficiency than the pure Pt loading Cu_2_ZnSnS_4_ [12]. Pt_3_Co bimetallic cocatalyst decorated CdS were prepared by Hu et al. and exhibited enhanced hydrogen evolution performance [13]. PtCo and/or PtFe loading Zn_1 − *x*_Cd_*x*_S were also evaluated in previous studies [14, 15]. However, low visible-light photocatalytic performance of Cu_2_ZnSnS_4_ or high toxicity of Cd hinders their practical application on a large scale. Carbon nitride (g-C_3_N_4_) has attracted attention due to its low cost [16]. Han et al. reported that a H_2_ evolution rate of 960 μmol g^−1^ h^−1^ was obtained over the PtCo/g-C_3_N_4_ photocatalyst under *λ* > 400 nm irradiation [17]. PtNi_x_/g-C_3_N_4_ hybird photocatalyst was also studied by Bi et al., and a H_2_ evolution rate of 8456 μmol g^−1^ h^−1^ was achieved under full spectral irradiation [18]. However, the visible-light photocatalytic performance for the bimetallic alloy cocatalyst-modified g-C_3_N_4_ photocatalyst is still a little bit low due to the wide band gap of 2.7 eV and bad electron transfer ability. Eosin Y-sensitized g-C_3_N_4_ can harvest a wide range of visible light [19, 20]. Graphene oxide (GO) possesses highly electron transporting property and has been widely used as an electron acceptor [21–25]. Combining g-C_3_N_4_ and GO can promote the electron transfer capability in g-C_3_N_4_ and thus improve the electron-hole pair’s separation to improve the photocatalytic performance for hydrogen evolution [26–31]. Lately, we reported an efficient Eosin Y-sensitized g-C_3_N_4_/Pt/GO composite photocatalyst for hydrogen evolution [23]. The expensive Pt cocatalyst plays one of the important role for the relatively high hydrogen production performance. In order to decrease the expensive Pt usage and further improve its visible photocatalytic performance, exploiting low-cost Eosin Y-sensitized g-C_3_N_4_/GO composite photocatalyst loaded with Pt-based alloy cocatalyst is useful.

Here, the Eosin Y-sensitized g-C_3_N_4_/PtNi/GO composite photocatalyst was prepared for hydrogen evolution from water. The highest hydrogen evolution rate of 5.89 mmol g^−1^ h^−1^ is obtained over the Eosin Y-sensitized g-C_3_N_4_/PtNi/GO photocatalyst, which is much higher than the Eosin Y-sensitized g-C_3_N_4_/Pt/GO and g-C_3_N_4_/Ni/GO composite samples. To the best our knowledge, there is no previous report that the Eosin Y-sensitized g-C_3_N_4_/PtNi/GO composite is employed for hydrogen production from water. The optimal molar ratio of Pt/Ni and amount of PtNi cocatalyst were screened out in detail. In addition, mechanism of enhanced photocatalystic performance for the g-C_3_N_4_/PtNi/GO composite was also investigated through different characterization methods.

## Experimental Section

### Synthesis of the g-C_3_N_4_

g-C_3_N_4_ powders were synthesized as described in previous study [32]. In a typical procedure, urea (8 g) was placed in an alumina crucible with a cover. The crucible was heated to 600 °C at a heating rate of 5 °C/min and held for 2 h in a tube furnace. After thermal treatment, the light yellow g-C_3_N_4_ powders were collected for further using.

### Preparation of GO

GO was prepared using the modified Hummers’ method [33]. Nature graphite (10 g) and NaNO_3_ (5 g) were putted into a beaker. Then, 230 mL concentrated sulfuric acid were added, and the process must be as slow as possible. The above reaction was proceeded with stirring under ice-water bath. Next, 10 g KMnO_4_ was added into the mixture solution and reacted for 3 h. The temperature of solution was raised to 35 °C and was maintained for 4 h. Then, 460-mL distilled water was poured into the above solution and heated up to about 98 °C for 3 h. After reaction, a certain amount of H_2_O_2_ (30%) and concentrated hydrochloric acid were added under stirring with the purpose of removing excess KMnO_4_ and SO_4_^2−^. Finally, the GO sample was obtained by freeze-drying for 24 h.

### Synthesis of g-C_3_N_4_/Ni/GO, g-C_3_N_4_/Pt/GO, and g-C_3_N_4_/Pt_x_Ni_y_/GO Composite Photocatalysts

Synthesis of g-C_3_N_4_/PtNi/GO-*X* (*X* represents the weight ratio of PtNi cocatalyst to g-C_3_N_4_/GO composite and the molar ratio of Pt to Ni is 1:1): In a typical, 133 mg of g-C_3_N_4_ were dispersed into 50 mL anhydrous ethanol. Excess NaBH_4_ reductant was added into the mixture solution under stirring. Then, a certain volume of NiCl_2_·6H_2_O solution (0.1 mol/L) and H_2_PtCl_6_ solution (1.0 mmol/L) were dropwise added into the above solution. In order to investigate the added procedure of NiCl_2_·6H_2_O and H_2_PtCl_6_ solutions, three methods including simultaneous loading of Pt and Ni, loading of Pt and then Ni or in reverse were chose. Then, the suspension solution was stirred for 5 h to achieve a uniform dispersion for PtNi cocatalyst in the g-C_3_N_4_. After that, the g-C_3_N_4_/PtNi-X samples were collected by centrifugation to wash away excess NaBH_4_ for several times. Afterwards, 67 mg GO and the g-C_3_N_4_/PtNi-X sample were dispersed into 100-mL distilled water simultaneously. The suspension solution was ultrasonicated at 500 W for 10 h. After that, a series of g-C_3_N_4_/PtNi/GO-*X* composite photocatalysts were obtained by centrifuged and then dried at 60 °C in a vacuum oven for one night. Other bimetallic PtNi alloy cocatalysts with different Pt/Ni molar ratio (9:1, 3:1, 1:3, 1:9) were also prepared as same as the aforementioned method, which named as Pt_9_Ni_1_, Pt_3_Ni_1_, Pt_1_Ni_3_, and Pt_1_Ni_9_, respectively, specially, the 1:1 M ratio of Pt/Ni name as PtNi.

Synthesis of g-C_3_N_4_/Ni/GO-0.5% and g-C_3_N_4_/Pt/GO-0.5% samples (0.5% represents the weight ratio of Ni or Pt to the g-C_3_N_4_/GO composite): The g-C_3_N_4_/Ni/GO-0.5% and g-C_3_N_4_/Pt/GO-0.5% samples were prepared with the same preparation procedure of g-C_3_N_4_/PtNi/GO-X samples except for adding different volume of NiCl_2_·6H_2_O solution or H_2_PtCl_6_ solution. The 2:1 weight ratio of g-C_3_N_4_ to GO is chosen in all g-C_3_N_4_/Ni/GO, g-C_3_N_4_/Pt/GO, and g-C_3_N_4_/Pt_x_Ni_y_/GO composite samples according to our previous study [32].

### Characterization Methods

The XRD patterns were obtained using an X-ray diffraction diffractometer (Bruker D8-Advance, Germany) with Cu-Kα radiation. TEM images of the samples were recorded through the transmission electron microscopy (JEM-2100, Japan). Surface chemical states of the photocatalysts were measured by X-ray photo-electron spectroscopy (XPS, AXISULTRA) with monochromatic Al Ka X-rays (1486.6 eV). The photoluminescence (PL) spectra were measured on a JY HORIBA FluoroLog-3 spectrometer, and the excited wavelength of 460 nm was chosen. The curves of photocurrent response were carried out in an electrochemical workstation (CHI660E, Chenhua, China) using a conventional standard three-electrode cell under visible light irradiation (λ > 420 nm). 0.1 mol/L Na_2_SO_4_ solution was used as electrolyte.

### Photocatalytic Activity Measurement

Photocatalytic experiments were conducted in a Pyrex cell with a top flat window at 6 °C. Typically, 50 mg photocatalyst powder and 50 mg Eosin Y dye were added into 100 mL H_2_O containing 20 vol% (*v*/v) triethanolamine (TEOA, pH = 7). A 300-W xenon lamp (D59, Beijing China Education Au-light Co., Ltd) coupled with a UV cut-off filter (> 420 nm) was used as the light source. The amounts of hydrogen were measured through a gas chromatography (GC-7920, TCD, Ar carrier).

## Results and Discussion

**Fig. 1:**
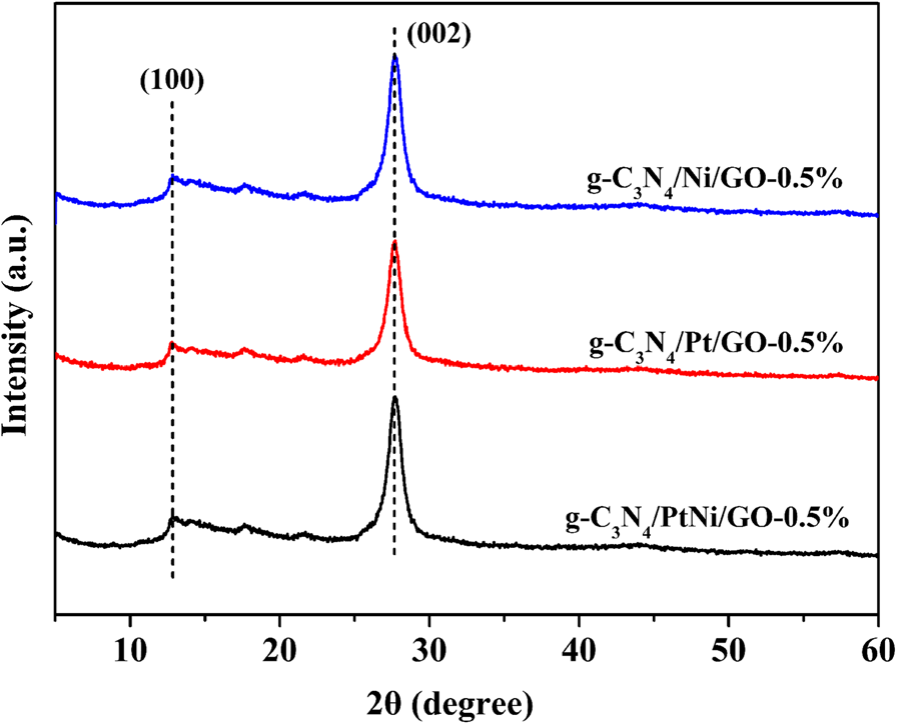
The XRD patterns of g-C_3_N_4_/Ni/GO-0.5%, g-C_3_N_4_/Pt/GO-0.5%, and g-C_3_N_4_/PtNi/GO-0.5% samples

**Fig. 2:**
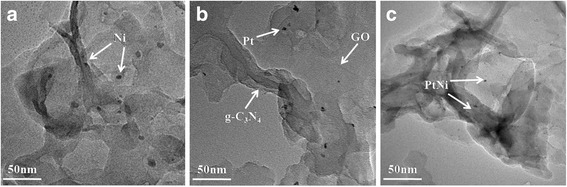
TEM images of g-C_3_N_4_/Ni/GO-0.5% (**a**), g-C_3_N_4_/Pt/GO-0.5% (**b**), and g-C_3_N_4_/PtNi/GO-0.5% (**c**)

The XRD patterns of g-C_3_N_4_/Ni/GO-0.5%, g-C_3_N_4_/Pt/GO-0.5%, and g-C_3_N_4_/PtNi/GO-0.5% samples are shown in Fig. 1. Two obvious diffraction peaks are observed for the three samples. A small peak centered at 2θ = 13.8° is assigned to the (100) peak of g-C_3_N_4_, which arises from the in-plane structural packing motif [34]. The strong diffraction peak at 27.4° is indexed as the (002) peak of g-C_3_N_4_, which corresponds to the interlayer stacking of conjugated aromatic system [35]. For the three evaluated samples, no GO, Pt and/or Ni cocatalyst diffraction signal were detected. Sufficient exfoliation of GO in the composite can result in no GO information [28]. While the amount of Pt and/or Ni are too small to detect by XRD method. As shown in Fig. 2, the three different samples exhibit similar thinner laminar structure after ultrasonic treatment. An intimate contact formed between 2D-layered structure of g-C_3_N_4_ and the nanosheet structure of GO. In Fig. 2a, b, a bit larger of Ni or Pt nanoparticles are dispersed on the interlayer or surface of g-C_3_N_4_. Compared with pure Ni and Pt, the size of the PtNi alloy cocatalyst particles is reduced and the dispersion of PtNi alloy cocatalyst is improved (see Fig. 2c). The small size of PtNi alloy cocatalyst will provide more reactive sites for hydrogen evolution, and the high dispersion of PtNi alloy cocatalyst is benefited to the electrons transfer from g-C_3_N_4_ and/or GO to PtNi cocatalyst. The improved dispersion of PtNi alloy cocatalyst was also observed in previous study [36]. The precision reason need further investigate.

**Fig. 3:**
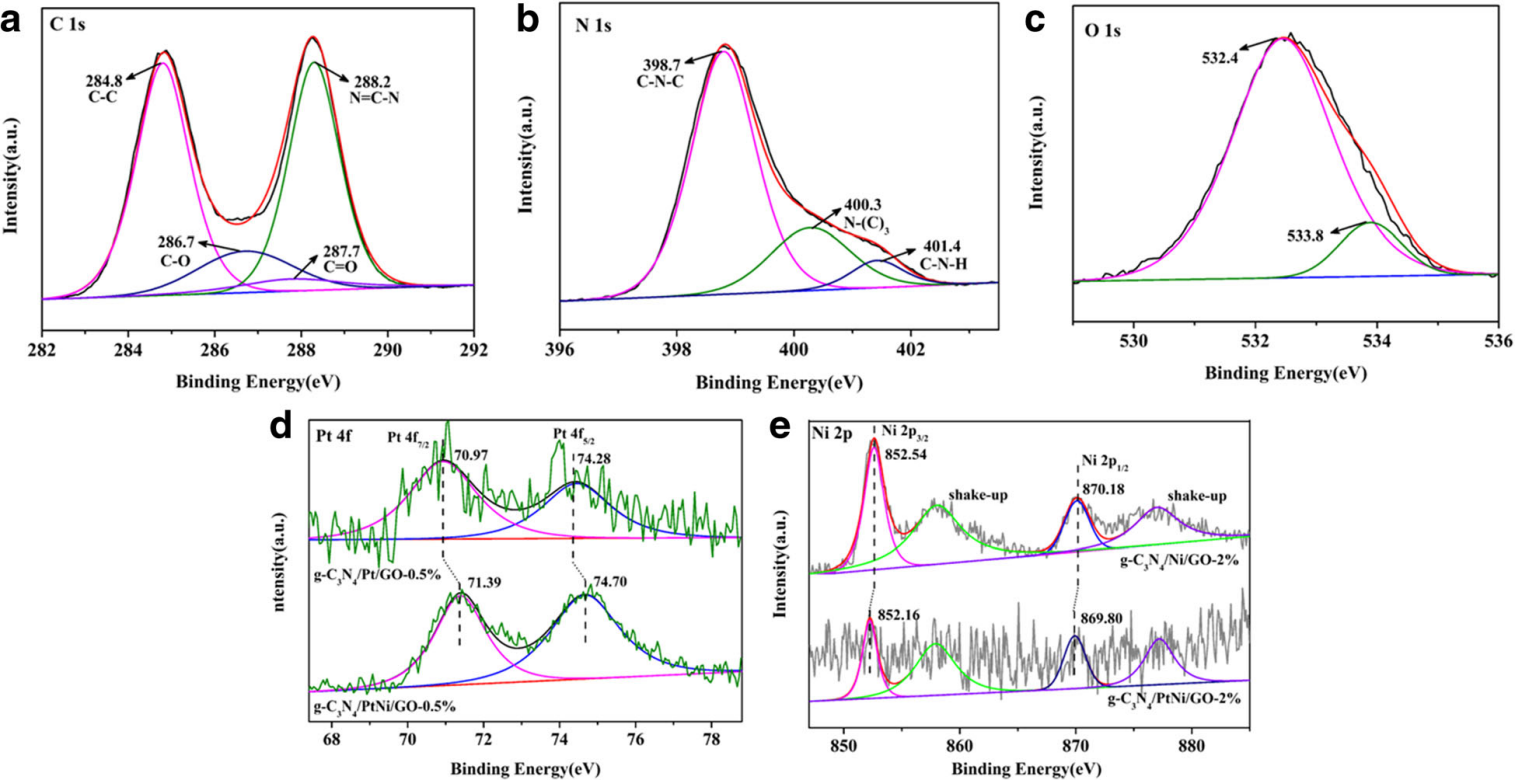
XPS spectra of (**a**) C 1 s, (**b**) N 1 s and (**c**) O 1 s for the g-C_3_N_4_/PtNi/GO-0.5% sample. **d** XPS spectra of Pt 4f for the g-C_3_N_4_/Pt/GO-0.5% and g-C_3_N_4_/PtNi/GO-0.5% samples. **e** XPS spectra of Ni 2p for the g-C_3_N_4_/Ni/GO-2% and g-C_3_N_4_/PtNi/GO-2% samples

In order to investigate the surface chemical element and valence states of g-C_3_N_4_/Ni/GO, g-C_3_N_4_/Pt/GO, and g-C_3_N_4_/PtNi/GO samples, high-resolution XPS spectra of the three different samples were measured and the results are shown in Fig. 3. In Fig. 3a, the XPS spectrum of C 1 s can be fitted into two strong peaks with the binding energies at about 284.8 eV and 288.2 eV, which are assigned to C-C and N=C-N, respectively [37]. The two peaks are the characteristic of carbon species in g-C_3_N_4_. Two small peaks at 286.7 eV and 287.7 eV are also obtained, which belong to the C-O and C=O functional groups on the surface of GO, respectively [38]. In Fig. 3 (b), the characteristic peaks of C-N-C, N-(C)_3_, and C-N-H groups in g-C_3_N_4_ were detected, which are located at the binding energies of 398.7, 400.3, and 401.4 eV, respectively [39]. In Fig. 3 (c), the banding energies of O 1 s are found at 532.4 and 533.8 eV, which are assigned to oxygen-containing functional groups in the composite sample and surface adsorption of oxygen species, respectively [40]. Figure 3 (d) exhibits the XPS spectra of the Pt 4f doublet (4f_7/2_ and 4f_5/2_). The Pt 4f_7/2_ and 4f_5/2_ peaks are located at 70.97 and 74.28 eV for the g-C_3_N_4_/Pt/GO-0.5% sample, respectively, which represent the signal of Pt^0^ [41, 42]. For the g-C_3_N_4_/PtNi/GO-0.5% sample, the orbital binding energies of Pt 4f shift about 0.42 eV to high binding energy compared with pure Pt. The obvious peak shift suggests that Pt electron is slight loss, which indicates that the PtNi alloy cocatalyst is formed in the g-C_3_N_4_/PtNi/GO-0.5% sample. As shown in Fig. 3e, the binding energies at 852.54 and 870.18 eV can be assigned to Ni 2p_3/2_ and Ni 2p_1/2_ for the g-C_3_N_4_/Ni/GO-2% sample, respectively, which are the characterization signal of Ni^0^ [43]. Compared with g-C_3_N_4_/Ni/GO-2%, the binding energies of Ni 2p shifted to low binding energy for the g-C_3_N_4_/PtNi/GO-2% sample. The result suggests that a change in the surrounding environment of Ni atoms occurs, which further confirms that the PtNi alloy cocatalyst is successfully synthesized [41]. The exact molar ratio of Pt to Ni in the g-C_3_N_4_/PtNi/GO-0.5% sample is 9:11 through XPS measurement. Based on above analysis, it can be concluded that the g-C_3_N_4_/PtNi/GO composite with PtNi alloy as cocatalyst was obtained by combing a facile liquid-phase sonochemical way with a chemical reduction method.

**Fig. 4:**
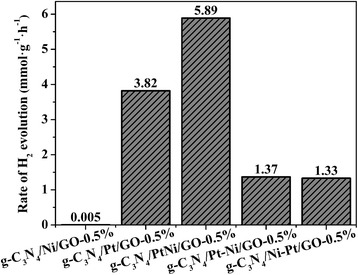
H_2_ evolution rate of series of g-C_3_N_4_/PtNi/GO-0.5% samples loaded with different type of cocatalyst. Light source: 300 W Xenon lamp (*λ* > 420 nm). Reaction solution: 100 mL 20% (*v*/v) TEOA aqueous solution (pH = 7)

Figure 4 shows the H_2_ evolution rate of series of g-C_3_N_4_/PtNi/GO-0.5% samples loaded with different type of cocatalyst. Simultaneous loading of Pt and Ni names as g-C_3_N_4_/PtNi/GO-0.5%; Loading of Pt and then Ni names as g-C_3_N_4_/Pt-Ni/GO-0.5%; Loading of Ni and then Pt names as g-C_3_N_4_/Ni-Pt/GO-0.5%. For Eosin Y-sensitized g-C_3_N_4_/Ni/GO-0.5% sample with pure Ni as cocatalyst, the H_2_ evolution rate is very low and only reach to 0.005 mmol g^−1^ h^−1^. After replacing Ni with Pt as cocatalyst, a significant increase of H_2_ evolution rate is observed, which sharply increases to 3.82 mmol g^−1^ h^−1^ for the Eosin Y-sensitized g-C_3_N_4_/Pt/GO-0.5% sample. The result suggests that loading efficient Pt cocatalyst is necessary to achieve excellent performance for H_2_ generation. When employed PtNi alloy as cocatalyst, the Eosin Y-sensitized g-C_3_N_4_/PtNi/GO-0.5% composite shows the highest H_2_ evolution rate of 5.89 mmol g^−1^ h^−1^, which is about 1.54 and 1178 times higher than the Eosin Y-sensitized g-C_3_N_4_/Pt/GO-0.5% and g-C_3_N_4_/Ni/GO-0.5% samples, respectively. The enhanced performance can be attributed to the positive synergistic effect between Pt and Ni. Compared with pure Pt cocatalyst, PtNi alloy cocatalyst accelerates the accumulation of photongenerated electrons, which will provide more electrons for hydrogen evolution [18]. In addition, the small size and high dispersion of PtNi alloy cocatalyst can provide more H_2_ evolution sites and enhance the electrons transfer, respectively. When loaded Pt and then Ni or in reverse, an obvious reduction of H_2_ evolution activity is observed. In fact, loading Pt and then Ni or in reverse does not form PtNi alloy cocatalyst [44]. The result indicates that achieving high H_2_ generation rate is strongly relied on employing efficient PtNi alloy cocatalyst.

**Fig. 5:**
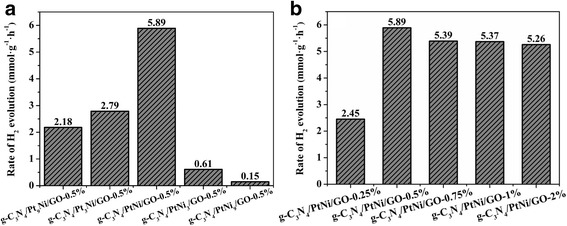
**a** H_2_ production rate of g-C_3_N_4_/Pt_x_Ni_y_/GO-0.5% samples loaded with different Pt/Ni molar ratio. **b** H_2_ production rate of g-C_3_N_4_/PtNi/GO samples loaded with different amounts of PtNi alloy cocatalyst. Light source: 300 W Xenon lamp (*λ* > 420 nm). Reaction solution: 100 mL 20% (*v*/*v*) TEOA aqueous solution (pH = 7)

**Fig. 6:**
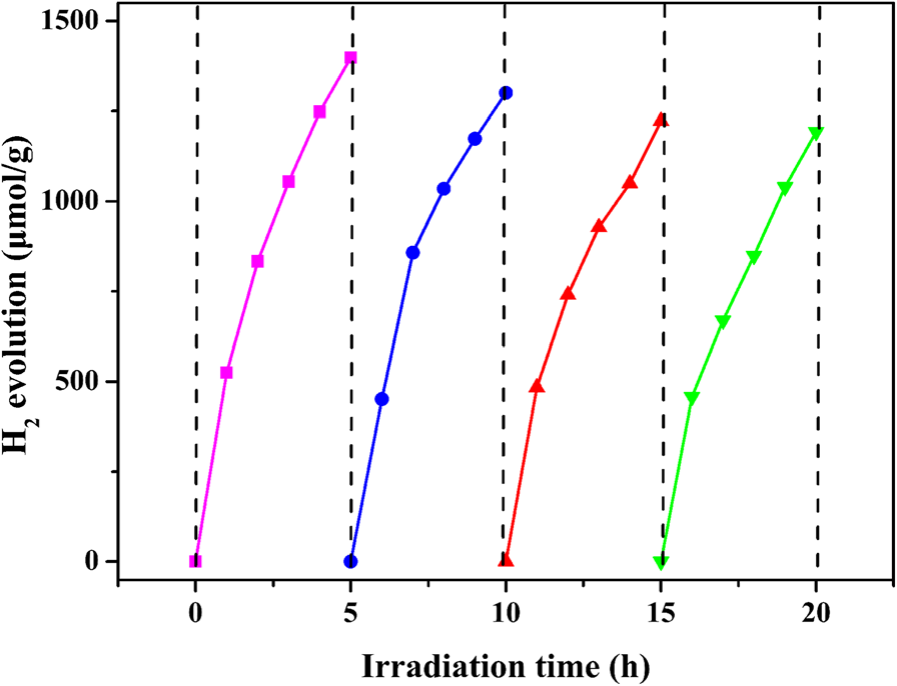
Cyclic H_2_ production for the g-C_3_N_4_/PtNi/GO-0.5% sample. Light source: 300 W Xenon lamp (λ > 420 nm), Reaction solution: 100 mL 20% (v/v) TEOA aqueous solution (pH = 7), photocatalyst, 50 mg, the weight ratio of Eosin Y to Photocatalyst is 1:1

The composition of PtNi alloy cocatalyst has an important effect on the catalytic activity for H_2_ evolution. Therefore, the H_2_ production rate of Eosin Y-sensitized g-C_3_N_4_/Pt_x_Ni_y_/GO-0.5% samples loaded with different Pt/Ni molar ratio were investigated and the results are shown in Fig. 5a. The H_2_ production rate is increased gradually with increasing Ni/Pt molar ratio. When the molar ratio of Ni/Pt is 1:1, the highest hydrogen production rate of 5.89 mmol g^−1^ h^−1^ is obtained. Further increasing the amount of Ni leads to a drop in the H_2_ evolution activity. The degradation of H_2_ generation performance may come from the reducing number of Pt active sites for H_2_ evolution. Pt active sites have stronger adsorption of hydrogen ions than Ni [12]. Therefore, H_2_ evolution preferentially takes places on Pt instead of Ni. Figure 5b shows the H_2_ production rate of Eosin Y-sensitized g-C_3_N_4_/PtNi/GO samples loaded with different amounts of PtNi alloy cocatalyst. When the weight content of PtNi alloy cocatalyst is 0.5%, a hydrogen evolution rate of 2.45 mmol g^−1^ h^−1^ is obtained for the g-C_3_N_4_/PtNi/GO-0.25% sample. The hydrogen evolution rate increases from 2.45 mmol g^−1^ h^−1^ to the highest value of 5.89 mmol g^−1^ h^−1^ after the amount of PtNi alloy cocatalyst up to 0.5%. In further increasing the amount of PtNi alloy cocatalyst, the hydrogen evolution performance shows a slight decrease. Excess PtNi alloy cocatalyst can hinder the light absorption of Eosin Y and g-C_3_N_4_ and thus degrade the photocatalytic performance. The stability of hydrogen generation for the g-C_3_N_4_/PtNi/GO-0.5% sample was also measured and the result is shown in Fig. 6. After 4 cycle test, the hydrogen generation rate for the g-C_3_N_4_/PtNi/GO-0.5% sample shows a slight decrease, which indicates that the g-C_3_N_4_/PtNi/GO-0.5% composite sample has relatively stability for hydrogen evolution. A strong covalent bond between the carbon and nitride in C_3_N_4_ and the weak degradation of Eosin Y are the two main reasons for the hydrogen evolution stability of g-C_3_N_4_/PtNi/GO-0.5% sample [19, 45].

**Fig. 7:**
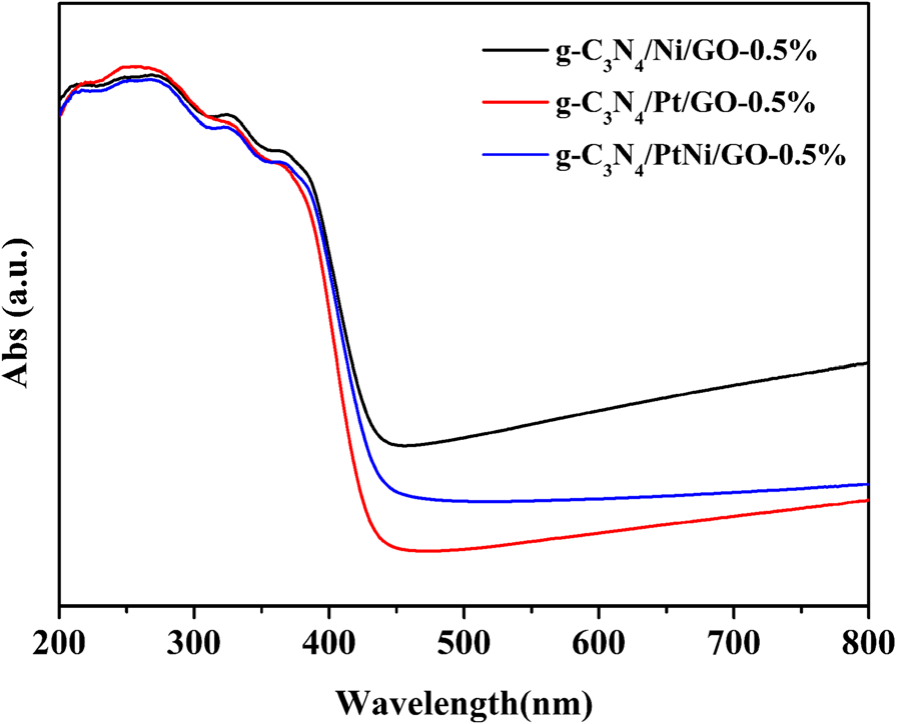
UV-Vis diffuse reflectance spectra of g-C_3_N_4_/Ni/GO-0.5%, g-C_3_N_4_/Pt/GO-0.5%, and g-C_3_N_4_/PtNi/GO-0.5% samples

In order investigate mechanism for the improved photocatalystic performance of g-C_3_N_4_/PtNi/GO sample with PtNi alloy as cocatalyst, two possible reasons of light absorption and charge separation efficiency were evaluated. Figure 7 shows the UV-Vis diffuse reflectance spectra of g-C_3_N_4_/Ni/GO-0.5%, g-C_3_N_4_/Pt/GO-0.5%, and g-C_3_N_4_/PtNi/GO-0.5% samples. The three different samples exhibit an obvious absorption after about 450 nm, which is from the metal cocatalyst [18]. The g-C_3_N_4_/Ni/GO-0.5% sample with pure Ni as cocatalyst shows the strongest absorption after about 450 nm. However, the H_2_ evolution rate for the g-C_3_N_4_/Ni/GO-0.5% sample is the lowest. The result suggests that the improved H_2_ evolution performance is not coming from the enhanced light absorption.

**Fig. 8:**
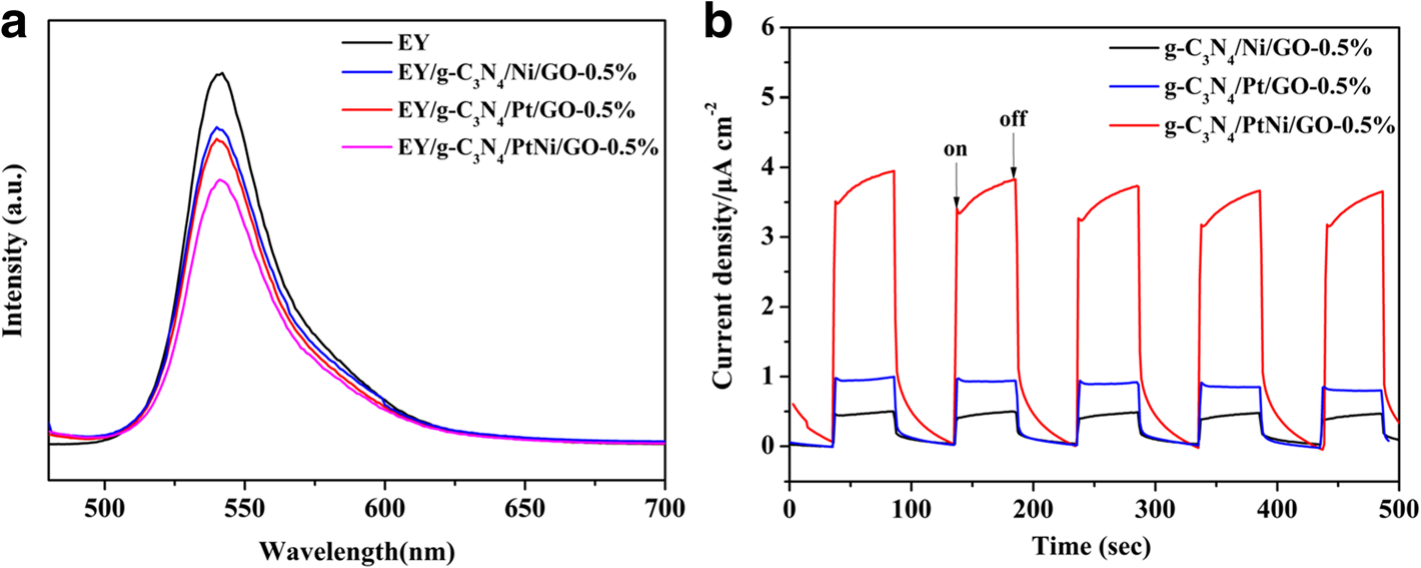
**a** The photoluminescence (PL) quenching spectra by g-C_3_N_4_/Ni/GO-0.5%, g-C_3_N_4_/Pt/GO-0.5% and g-C_3_N_4_/PtNi/GO-0.5% samples in 20% (v/v) TEOA aqueous solution (pH = 7). Eosin Y solution: 0.01 mM (**b**) Transient photocurrent responses of g-C_3_N_4_/Ni/GO-0.5%, g-C_3_N_4_/Pt/GO-0.5% and g-C_3_N_4_/PtNi/GO-0.5% samples

Charge separation efficiency can be characterized by the photoluminescence (PL) quenching spectra [46, 47]. In general, a strong intensity of PL spectra indicates serious charge carrier recombination. Figure 8a shows the photoluminescence (PL) quenching spectra of Eosin Y by the g-C_3_N_4_/Ni/GO-0.5%, g-C_3_N_4_/Pt/GO-0.5%, and g-C_3_N_4_/PtNi/GO-0.5% samples. Only the Eosin Y solution without photocatalyst exhibits an extensive emission peak at about 540 nm because of Eosin Y’s conjugate xanthene structure and strong recombination capacity of photogenerated electron-hole pairs in excited Eosin Y. Obvious fluorescence quenching is observed after adding different type of photocatalysts into the Eosin Y solution. The fluorescence quenching suggests that the electrons transfer to photocatalysts from excited Eosin Y and then migrate to cocatalyst for proton reduction. In addition, there is a small blue shift (ca. 1.3 nm) of PL quenching spectra for the three different evaluated composite photocatalysts, which can be attributed to the noncovalent π-π stacking interaction among Eosin Y, g-C_3_N_4_ and GO [48]. The PL quenching spectra of g-C_3_N_4_/Pt/GO-0.5% sample shows a moderated intensity, which is lower than the g-C_3_N_4_/Ni/GO-0.5% sample. Importantly, the g-C_3_N_4_/PtNi/GO-0.5% sample exhibits the lowest fluorescence intensity, which implies that the PtNi alloy cocatalyst is the most efficient cocatalyst for improving the charge separation efficiency than pure Pt or Ni. The result is consistent with the H2 evolution activity (see Fig. 4). To further verify the charge transfer process, the transient photocurrent responses of g-C_3_N_4_/Ni/GO-0.5%, g-C_3_N_4_/Pt/GO-0.5%, and g-C_3_N_4_/PtNi/GO-0.5% samples were measured and the results are shown in Fig. 8b. The g-C_3_N_4_/PtNi/GO-0.5% sample exhibits the highest photocurrent response under visible-light irradiation (λ > 420 nm), which further confirms employing PtNi alloy cocatalyst is essential to improve charge separation efficiency. Based on above results, the improved H2 evolution activity for the Eosin Y-sensitized g-C_3_N_4_/PtNi/GO-0.5% composite is attributed to the enhanced charge separation efficiency.

**Fig. 9:**
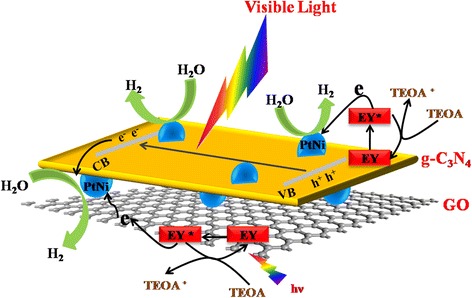
A schematic diagram of H_2_ evolution process for the Eosin Y-sensitized g-C_3_N_4_/PtNi/GO composite sample under visible light irradiation

According to above results and mechanism analysis, we propose a schematic diagram to understand the H_2_ evolution process for the Eosin Y-sensitized g-C_3_N_4_/PtNi/GO composite sample (Fig. 9). Under visible-light irradiation, the photogenerated electrons in the LUMO of excited Eosin Y transfer to g-C_3_N_4_ and/or GO and then to the PtNi alloy cocatalyst for protons reduction. Meanwhile, the photoexcited electrons in the CB of g-C_3_N_4_ also flow to the PtNi alloy cocatalyst for H_2_ evolution reaction. At the same time, the photogenerated holes or oxidized Eosin Y dyes directly oxide the TEOA sacrificial agent.

## Conclusion

Ternary g-C_3_N_4_/PtNi/GO composite was synthesized by combing a facile liquid-phase sonochemical way with a chemical reduction method. The Eosin Y-sensitized g-C_3_N_4_/PtNi/GO-0.5% composite shows the highest hydrogen evolution rate of 5.89 mmol g^−1^ h^−1^, which is about 1.54 and 1178 times higher than the Eosin Y-sensitized g-C_3_N_4_/Pt/GO-0.5% and g-C_3_N_4_/Ni/GO-0.5% samples, respectively. The enhanced photocatalytic activity can be ascribed to the positive synergetic effect between Pt and Ni as well as more reactive sites, which leads to efficient photoexcited electron-hole pairs separation. This study demonstrates that PtNi alloy can be employed as an economic and efficient cocatalyst for photocatalytic hydrogen evolution.
